# Impact of the COVID-19 Pandemic on the Implementation of Mobile Health to Improve the Uptake of Hydroxyurea in Patients With Sickle Cell Disease: Mixed Methods Study

**DOI:** 10.2196/41415

**Published:** 2022-10-14

**Authors:** Sherif M Badawy, Lisa DiMartino, Donald Brambilla, Lisa Klesges, Ana Baumann, Ebony Burns, Terri DeMartino, Sara Jacobs, Hamda Khan, Chinonyelum Nwosu, Nirmish Shah, Jane S Hankins, Sickle Cell Disease Implementation Consortium

**Affiliations:** 1 Division of Hematology Oncology and Stem Cell Transplant Ann & Robert H. Lurie Children's Hospital of Chicago Chicago, IL United States; 2 Department of Pediatrics Northwestern University Feinberg School Medicine Chicago, IL United States; 3 RTI International Research Triangle Park, NC United States; 4 Department of Surgery Washington University in St. Louis St. Louis, MO United States; 5 Division of Hematology Duke University School of Medicine Durham, NC United States; 6 Department of Hematology St. Jude Children's Research Hospital Memphis, TN United States; 7 University of Tennessee Health Science Center Memphis, TN United States; 8 See Acknowledgments

**Keywords:** sickle cell anemia, implementation science, adherence, hydroxycarbamide, self-efficacy

## Abstract

**Background:**

Hydroxyurea therapy is effective for reducing complications related to sickle cell disease (SCD) and is recommended by National Health Lung and Blood Institute care guidelines. However, hydroxyurea is underutilized, and adherence is suboptimal. We wanted to test a multilevel mobile health (mHealth) intervention to increase hydroxyurea adherence among patients and improve prescribing among providers in a multicenter clinical trial. In the first 2 study sites, participants were exposed to the early phases of the COVID-19 pandemic, which included disruption to their regular SCD care.

**Objective:**

We aimed to describe the impact of the COVID-19 pandemic on the implementation of an mHealth behavioral intervention for improving hydroxyurea adherence among patients with SCD.

**Methods:**

The first 2 sites initiated enrollment 3 months prior to the start of the pandemic (November 2019 to March 2020). During implementation, site A clinics shut down for 2 months and site B clinics shut down for 9 months. We used the reach, effectiveness, adoption, implementation, and maintenance (RE-AIM) framework to evaluate the implementation and effectiveness of the intervention. mHealth implementation was assessed based on patients’ daily app use. Adherence to hydroxyurea was calculated as the proportion of days covered (PDC) from prescription records over the first 12 and 24 weeks after implementation. A linear model examined the relationship between app usage and PDC change, adjusting for baseline PDC, lockdown duration, and site. We conducted semistructured interviews with patients, health care providers, administrators, and research staff to identify factors associated with mHealth implementation and effectiveness. We used a mixed methods approach to investigate the convergence of qualitative and quantitative findings.

**Results:**

The percentage of patients accessing the app decreased after March 15, 2020 from 86% (n=55) to 70% (n=45). The overall mean PDC increase from baseline to week 12 was 4.5% (*P*=.32) and to week 24 was 1.5% (*P*=.70). The mean PDC change was greater at site A (12 weeks: 20.9%; *P*=.003; 24 weeks: 16.7%; *P*=.01) than site B (12 weeks: −8.2%; *P*=.14; 24 weeks: −10.3%; *P*=.02). After adjustment, PDC change was 13.8% greater in those with increased app use after March 15, 2020. Interview findings indicated that site B’s closure during COVID-19 had a greater impact, but almost all patients reported that the *InCharge Health* app helped support more consistent medication use.

**Conclusions:**

We found significant impacts of the early clinic lockdowns, which reduced implementation of the mHealth intervention and led to reduced patient adherence to hydroxyurea. However, disruptions were lower among participants who experienced shorter clinic lockdowns and were associated with higher hydroxyurea adherence. Investigation of added strategies to mitigate the effects of care interruptions during major emergencies (eg, patient coaching and health navigation) may “insulate” the implementation of interventions to increase medication adherence.

**Trial Registration:**

ClinicalTrials.gov NCT04080167; https://clinicaltrials.gov/ct2/show/NCT04080167

**International Registered Report Identifier (IRRID):**

RR2-10.2196/16319

## Introduction

Sickle cell disease (SCD) is a chronic blood disorder in which acute painful acute events occur on the background of progressive organ dysfunction, leading to premature mortality [1]. SCD disproportionately affects low-income Americans who face access barriers to evidence-based treatments [2]. Hydroxyurea therapy is effective in reducing SCD-related complications, including acute pain episodes, and is recommended by National Health Lung and Blood Institute (NHLBI) care guidelines [1,3] However, hydroxyurea is underutilized and adherence is suboptimal because providers often have a limited understanding of the optimal use of hydroxyurea [4,5], and many patients lack motivation and general knowledge about hydroxyurea and fear complications or side effects [6,7]. These barriers further increase health disparities in the care of the SCD population.

**Figure 1:**
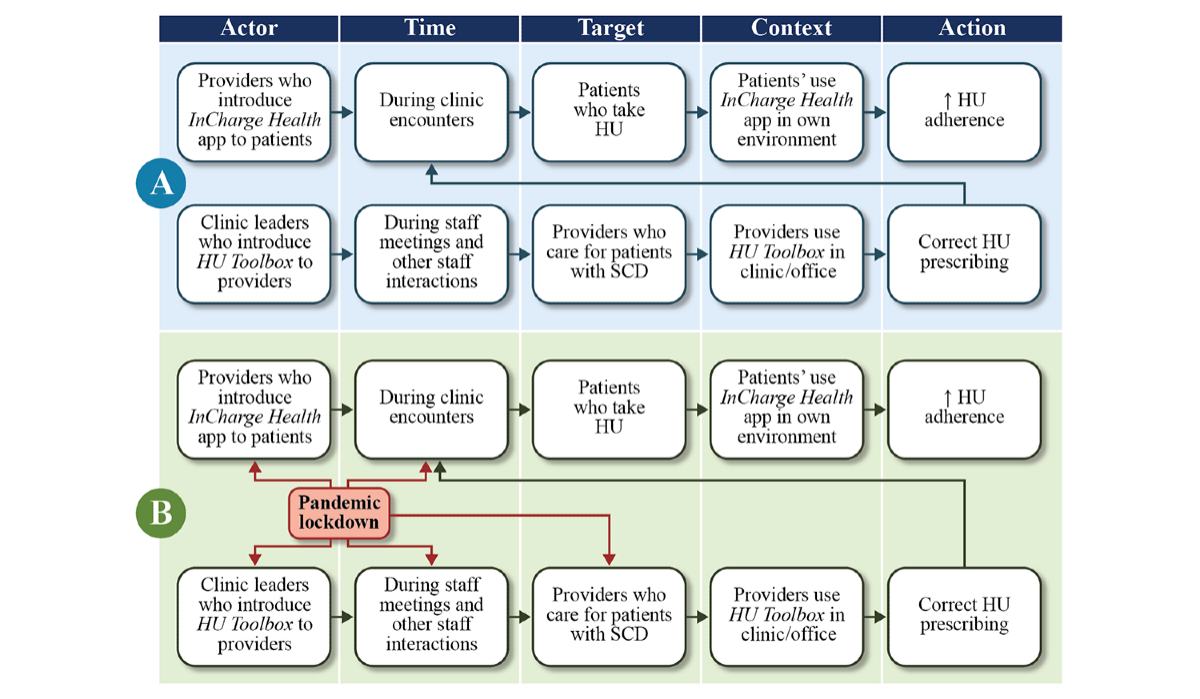
Implementation intervention specification. Specification is done according to the action, actor, context, target, and time (AACTT) framework [14]. (A) Flow before the COVID-19 pandemic. (B) After the start of the COVID-19 pandemic, clinic lockdown measures were put in place, which led to reductions in leadership-staff interactions and patient-provider interactions. Introduction of the respective apps and hydroxyurea (HU) prescribing were, thereby, reduced, leading to lower HU adherence among patients. SCD: sickle cell disease.

Mobile health (mHealth) apps, for both patients and providers, can be used as a strategy to incorporate behavioral change interventions that can potentially improve medication adoption and effectiveness [8-10]. Two months prior to when the COVID-19 pandemic reached US soil, we initiated a multicenter study as part of the NHLBI-funded Sickle Cell Disease Implementation Consortium (SCDIC) [11,12] to investigate the effectiveness and measure the implementation outcomes of a 2-level intervention using mHealth to support hydroxyurea adherence among patients (*InCharge* *Health* app) [13] and hydroxyurea prescribing among providers (*HU Toolbox* app). This multilevel strategy focused on increasing hydroxyurea use, by targeting the determinants involved in medication adherence (ie, motivation, knowledge, self-efficacy, and social support) among patients (*InCharge Health* app) and those involved in appropriate prescribing (eg, knowledge, attitude, and self-efficacy) among providers (*HU Toolbox* app). To facilitate the description and identification of the characteristics of the mHealth intervention, we specified them according to the action, actor, context, target, and time (AACTT) framework [14]. Providers (actor) introduce the *InCharge Health* app during clinic encounters (time) to patients (target), who then use the app in their own environment (context) to improve hydroxyurea adherence behavior (action) (Figure 1A). Clinic leaders (actor) introduce the *HU Toolbox* app to providers who care for patients with SCD (target) during clinic staff interactions (time), who then use the toolbox in their offices or clinics (context) to improve correct hydroxyurea prescribing behavior (action) (Figure 1A). In clinical practice, as providers prescribe and counsel patients on the benefits of hydroxyurea during regular visits, providers are both the actors and targets of our multilevel intervention.

The COVID-19 pandemic has disrupted health care access in unprecedented ways, including reducing patient-provider contact during health maintenance care and reducing medication adherence for chronic diseases [15-17]. In particular, many SCD patients are often seen in outpatient clinics every 2 to 4 months if they are on a disease-modifying therapy, such as hydroxyurea, or every 6 months if they are not. This close monitoring of their clinical condition and treatment plan was deeply affected by the COVID-19 pandemic. Additionally, patients with SCD had worse outcomes from COVID-19 infection compared with race-matched individuals without SCD [18]. Leveraging mHealth interventions to facilitate health care delivery, including the use of evidence-based hydroxyurea, is underscored by the COVID-19 pandemic [15]. However, how the pandemic may affect mHealth use for medication adherence in SCD has not been investigated and remains unclear. Hydroxyurea use can be potentially amplified by mHealth, but disruptions during the COVID-19 pandemic may threaten its implementation.

Because the COVID-19 pandemic lockdown restrictions disrupted the care of the study participants, we sought to evaluate how the implementation and preliminary effectiveness of the patient *InCharge Health* app were affected. We hope our lessons learned will inform future studies disrupted by unplanned emergencies, such as pandemics, by anticipating possible required study adaptations.

## Methods

### Study Setting and Participants

In this report, we describe the results of the implementation of the *InCharge Health* app in the first 2 (out of 7) SCDIC study sites, to represent the early impact of the pandemic response. The study has been registered at ClinicalTrials.gov (NCT04080167). Both sites were in the south region of the United States, and they initiated study enrollment 3 months prior to the start of the pandemic (November 2019 to March 2020) as part of a planned staggered intervention design [11]. As a response to the COVID-19 pandemic and to reduce virus spread, both sites temporarily suspended nonemergent in-person clinical activities. During implementation, site A clinics closed for 2 months (March 15, 2020, to May 15, 2020) and quickly initiated telemedicine visits, which were already standard practice at this institution, but were scaled up during the pandemic. Site B clinics were closed for 9 months (March 15, 2020, to December 15, 2020) and did not initiate telehealth visits until year 2 of the pandemic due to delays in training staff and distributing equipment. The 5 remaining sites started enrollment later in the pandemic when the clinic lockdowns were no longer in effect, and therefore, their results are not being reported here.

Patient participants were individuals with a diagnosis of SCD between the ages of 15 and 45 years treated with hydroxyurea and receiving care at the 2 initial participating sites [12]. Patients with SCD receiving chronic blood transfusions or using another mHealth modality for medication adherence were not eligible. To best represent the impact of both site’s pandemic response lockdown, only patient participants who enrolled prior to March 15, 2020, and were followed up until after March 15, 2020, were included in this analysis, allowing the contrast of their study behavior before and after the start of the pandemic. The date of enrollment varied among subjects, but follow-up time was the same for all subjects in this analysis (24 weeks). Therefore, the time over which subjects were exposed to the shutdown varied, a point that is considered in more detail below. Provider participants were physicians and advance care practitioners (nurse practitioners and physician assistants) who cared for at least 1 patient with SCD within the participating sites.

### Ethics Approval

This study was approved by the Institutional Review Board at St Jude Children's Research Hospital (19-0159) and Duke University (Pro00073506), and all participants (or their legal guardians) signed consent prior to study participation.

### Study Design and Key Measures

The methods of the study have been published [11]. Briefly, all patients enrolled were asked to download the *InCharge Health* app on their cell phones and use it for at least 24 weeks. Providers were asked to download the *HU Toolbox* app and use it for at least 9 months. Study visits occurred 12 and 24 weeks after enrollment, and data regarding app use and hydroxyurea refills were collected.

As part of the planned approach for the multisite study, we used the reach, effectiveness, adoption, implementation, and maintenance (RE-AIM) framework to inform the evaluation of the implementation and effectiveness of the *InCharge Health* app [19]. mHealth implementation was assessed based on user mHealth engagement, which was classified according to patients’ daily app use (number of days accessed during study participation). App use was categorized as follows: low (<25% days/month), medium-low (25%-49% days/month), medium-high (50%-74% days/month), and high (75%-100% days/month) [11].

Patient-level data on hydroxyurea adherence provided a measure of effectiveness. Hydroxyurea adherence was measured by calculating the proportion of days covered (PDC), which is the ratio of the number of days the patient is covered by the medication to the number of days in the treatment period. In other words, the PDC is the number of days covered by prescriptions that were filled divided by the length of the study interval. If a prescription was filled just before the start of the study interval, the days between the prescription fill date and start of the interval were excluded. If a prescription was filled near the end of the study interval, the part of the interval covered by the prescription that was after the end of the study interval was also excluded. The PDC was calculated over a 24-week baseline interval and over the first 12 and first 24 weeks after implementation. Provider app use was classified as low (≤1 time on average monthly during the study) and high (>1 time on average monthly during the study).

### Quantitative Analysis

We compared PDC change over 12 and 24 weeks after mHealth implementation using 1-sample *t* tests. The relation between the amount of app usage and PDC change was calculated using linear models of PDC change as a function of app use, treating app use as continuous in some models and as a 4-level categorical variable in others. The choice of linear models was based on prior experience showing that linear models were appropriate for analyzing changes in the PDC. To ensure the appropriateness of this method, we verified the fit of the models by examining the distributions of residuals and the relationships between residuals and predicted values. Site was included in some models to allow for variation among sites in changes in the PDC that might have been induced by site-to-site variations in the responses to the pandemic. By definition, the PDC is ≥0% and ≤100%. It is very common for change in a bounded measure, such as PDC, to be negatively correlated with the baseline value. Baseline PDC was therefore included in most models to avoid confounding between baseline PDC and other predictors of interest. The results are presented as the differences between changes in PDC at the 2 sites, adjusted for baseline PDC. While power calculations are not provided for this study, the large ongoing trial power calculation has been published [11]. The reach of the *InCharge Health* app was considered as the proportion of eligible patients who were enrolled in the study, and of those enrolled, the proportion who downloaded and used the app at least once. Adoption of *HU Toolbox* was considered as the proportion of eligible providers who were enrolled, and of those enrolled, the proportion who downloaded and used the app at least once. App use was measured as the proportion of follow-up days on which the app was accessed at least once per day. Use was calculated over the entire follow-up for each patient participant in some analyses and separately for the periods from enrollment up through March 15, 2020, and after March 15, 2020, in others. Given that the length of follow-up was the same for all subjects, the number of days from enrollment through March 15, 2020, was a measure of the proportion of the follow-up interval the subjects experienced before March 15, 2020 (ie, the start of the lockdown period). Logistic regression was employed to identify the predictors of increased app use after March 15, 2020, in those with follow-up time both before and after that date.

The association between the use of the *InCharge Health* app and PDC change from baseline through follow-up was examined in linear models of PDC change as a function of baseline PDC, time from March 15, 2020, to the end of each subject’s follow-up, an indicator for site, and app use during the follow-up interval. The following 2 measures of app use were considered in separate sets of models: (1) the number of follow-up days on which the app was accessed at least once and (2) a binary indicator for whether app use increased or decreased after March 15, 2020. Interaction terms were also considered, particularly interactions between site and the other predictors, to determine whether the effects of any predictors differed at the 2 sites.

### Qualitative Analysis

We used the RE-AIM framework to qualitatively identify factors that may have influenced mHealth implementation and effectiveness during the initial phases of the COVID-19 pandemic [20]. Semistructured interviews were conducted with patients to better understand the contextual factors associated with *InCharge Health* app implementation and effectiveness. Health care providers, administrators, and research staff were also interviewed to provide qualitative data regarding the impact of the COVID-19 pandemic on app use. Interviews were conducted by research coordinators between June 2020 and March 2021. Semistructured interview guides were developed using the RE-AIM framework to understand participants’ engagement and experiences with the apps, and included several questions specific to COVID-19 (Multimedia Appendix 1) [20,21]. For example, participants were asked to describe how using the app changed the way they took hydroxyurea during COVID-19 (effectiveness) and how COVID-19 impacted use of the app (implementation). For interviews with research staff, questions were asked about the challenges related to COVID-19 encountered at the site. A purposive sample of participants was interviewed based on app use frequency. Data were transcribed and entered into NVivo 12.0 (QSR International) for qualitative data analysis. Data were coded and analyzed with the goal of achieving theme saturation. Results were grouped into themes and mapped to the effectiveness and implementation RE-AIM domains by 2 study team members. We used mixed methods to investigate the possible convergence of qualitative and quantitative findings, through data triangulation [22]. Through this analysis, we sought to corroborate and expand quantitative findings using qualitative data.

## Results

### Participant Characteristics and Study Participation

A total of 75 patients (out of 508 eligible) and 42 providers (out of 55 eligible) were enrolled between November 2019 and September 2020 in the first 2 participating SCDIC sites. To characterize the influence of the COVID-19 pandemic shutdowns on app use, we only included 64 patients and all 42 providers enrolled prior to March 15, 2022. Among the patients, 28 were enrolled at site A and 36 at site B. Half (32/64, 50%) were young adults (18-25 years), with almost even gender distribution (Table 1). Among the providers, most (29/42, 69%) were between 26 and 45 years of age, and majority were female (30/42, 71%) and physicians (24/42, 59%) (Table 1)*.*

**Table 1:** Participant characteristics.

Characteristic			Patients, n (%)							Providers, n (%)				
			All patients (N=64)		Site A (N=28)		Site B (N=36)		All providers (N=42)			Site A (N=15)		Site B (N=27)
**Age range (years)**														
	15-17	7 (11)		0 (0)		7 (19)		0 (0)			0 (0)		0 (0)	
	18-25	32 (50)		10 (36)		22 (61)		0 (0)			0 (0)		0 (0)	
	26-45	25 (39)		18 (64)		7 (19)		29 (69)			9 (60)		20 (74)	
	46-64	0 (0)		0 (0)		0 (0)		12 (29)			5 (33)		7 (26)	
	>65	0 (0)		0 (0)		0 (0)		0 (0)			0 (0)		0 (0)	
	Missing	0 (0)		0 (0)		0 (0)		1 (2)			1 (7)		0 (0)	
**Gender**														
	Male	31 (48)		15 (54)		16 (44)		12 (29)			2 (13)		10 (37)	
	Female	33 (52)		13 (46)		20 (56)		30 (71)			13 (87)		17 (63)	
**Race**														
	Black	64 (100)		28 (100)		36 (100)		8 (19)			5 (33)		3 (11)	
	White	0 (0)		0 (0)		0 (0)		24 (59)			7 (47)		17 (63)	
	Asian	0 (0)		0 (0)		0 (0)		9 (22)			2 (13)		7 (26)	
	Missing	0 (0)		0 (0)		0 (0)		1 (2)			1 (7)		0 (0)	
**Ethnicity**														
	Not Hispanic	64 (100)		28 (100)		36 (100)		41 (98)			14 (93)		27 (100)	
	Hispanic	0 (0)		0 (0)		0 (0)		1 (2)			1 (7)		0 (0)	
**Sickle cell disease genotype**														
	HbSS/HbSβ^0^-thalassemia	56 (88)		27 (96)		29 (81)		N/A^a^			N/A		N/A	
	HbSC/HbSβ^+^-thalassemia/other	8 (12)		1 (4)		7 (19)		N/A			N/A		N/A	
**Provider type**														
	Physician	N/A		N/A		N/A		24 (59)			9 (60)		15 (56)	
	Nurse practitioner or physician assistant	N/A		N/A		N/A		17 (41)			6 (40)		11 (41)	
	Missing	N/A		N/A		N/A		1 (2)			0 (0)		1 (4)	
***InCharge Health* app use level^b^**														
	High	6 (9)		6 (21)		0 (0)		N/A			N/A		N/A	
	Medium-high	8 (13)		5 (18)		3 (8)		N/A			N/A		N/A	
	Medium-low	9 (14)		2 (7)		7 (19)		N/A			N/A		N/A	
	Low	41 (64)		15 (54)		26 (72)		N/A			N/A		N/A	
***HU Toolbox* app use level^c,d^**														
	High	N/A		N/A		N/A		21 (52)			8 (53)		13 (48)	
	Low	N/A		N/A		N/A		19 (48)			5 (33)		14 (52)	

^a^N/A: not applicable.

^b^The *InCharge Health* app use level for patients was categorized based on the percentage of days used per month as follows: low, <25%; medium-low, 25%-50%; medium-high, 51%-74%; and high, 75%-100%.

^c^The *HU-Toolbox* app use level for providers was categorized as low (<1 app usage per month) and high (≥1 app usage per month) over a 9-month period.

^d^Two providers were removed from the study (moved to a new institution or requested to be withdrawn).

### App Use

**Figure 2:**
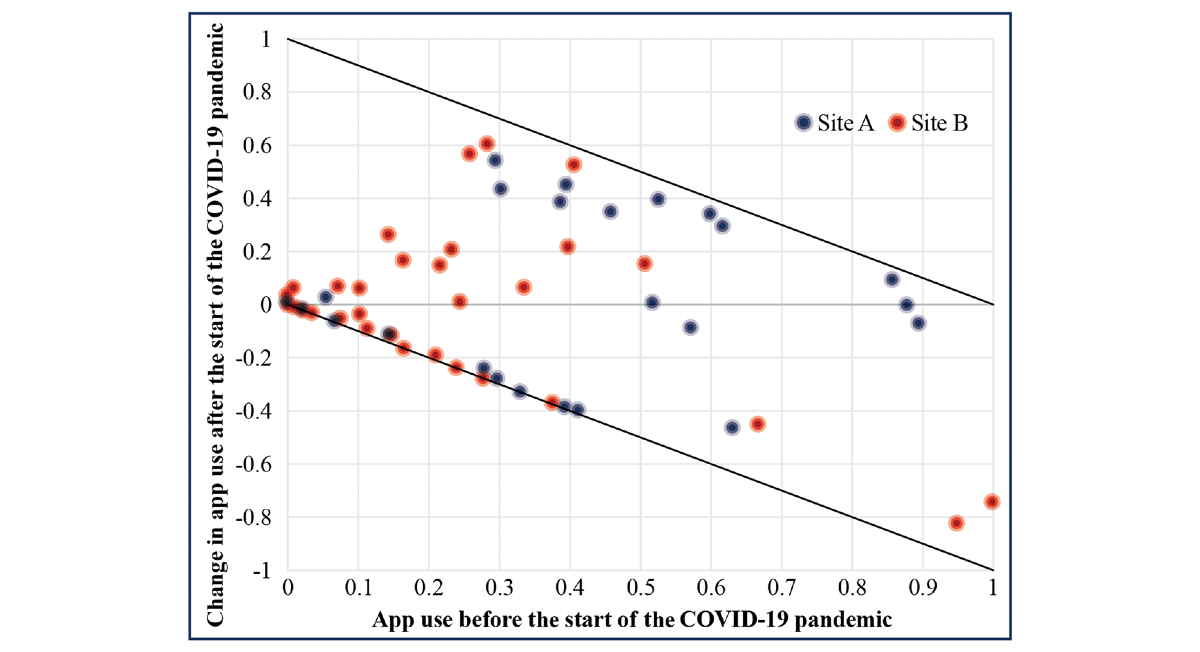
Change in InCharge Health app use relative to the COVID-19 pandemic lockdown. March 15, 2020, corresponds to the date when both sites went on lockdown in response to the COVID-19 pandemic. The black diagonal lines represent the boundaries for the maximum that app use can change after March 15, 2020, given app use before March 15, 2020. Since app use is expressed as a proportion of days on which the app is accessed, app use must be ≥0 and ≤1.0. As app use prior to March 15, 2020, increases, the maximum amount by which it can drop after March 15, 2020, increases, while the amount by which it can increase after March 15, 2020, decreases. For example, if app use is 0.25 (25% of days) before March 15, 2020, it can drop by a maximum of 0.25 or increase by a maximum of 0.75, whereas if app use is 0.75 (75% of days) before March 15, 2020, it can drop by a maximum of 0.75 or increase by a maximum of 0.25. There were 2 subgroups. The diagonal line of points along the lower black boundary line indicates the first subgroup consisting of participants whose app use dropped from some use to little or no use after March 15, 2020. On the other hand, the cloud of points from both sites above the line of zero change indicates the second subgroup consisting of patients whose app use increased after March 15, 2020.

All 64 patient participants downloaded the app, and 58 participants used it at least once during the 6-month study period, representing a 91% reach. On average, patients accessed the app on 42.7 (25.5%) days throughout the 6 months of the study period, and 24 (38%) of the 64 patients accessed it on ≥25% of the total days over 6 months. The percentage of participants accessing the app decreased after March 15, 2020, from 86% (n=55) before that date to 70% (n=45) after that date. However, the average change in app use was very close to 0 (mean change: −0.0016; *P*=.96), which means that reductions in use by some participants were balanced by increases in others. It is important to note, however, that there appeared to be 2 distinct subgroups at each site, one with increased app use after March 15, 2020, and the other with decreased app use after March 15, 2020 (Figure 2). A logistic regression of the probability of increasing app use after March 15, 2020, indicated that the probability decreased with increasing time between March 15, 2020, and the end of follow-up, but did not differ between sites (OR −0.0276; *P*=.004). The odds ratio for increased app use was 0.67 when the time from March 15, 2020, to the end of follow-up increased by 14.7 days, and it decreased to 0.5 when the time increased to 25 days. Thus, longer exposure to the shutdown was associated with a reduction in app use. Other predictors were considered, including demographic variables, such as gender, age, income, and education, and measures of pain frequency, pain intensity, and recent use of hydroxyurea, but none made statistically significant contributions to the model for increased app use after March 15, 2020.

Of the 42 providers enrolled, 41 downloaded and used the *HU Toolbox* app at least once (adoption 98%). Overall app use among providers averaged around a day per month (1.1 days per month) prior to the pandemic, but declined to less than a day per month (0.2 days per month) during the lockdown.

### Hydroxyurea Adherence

**Figure 3:**
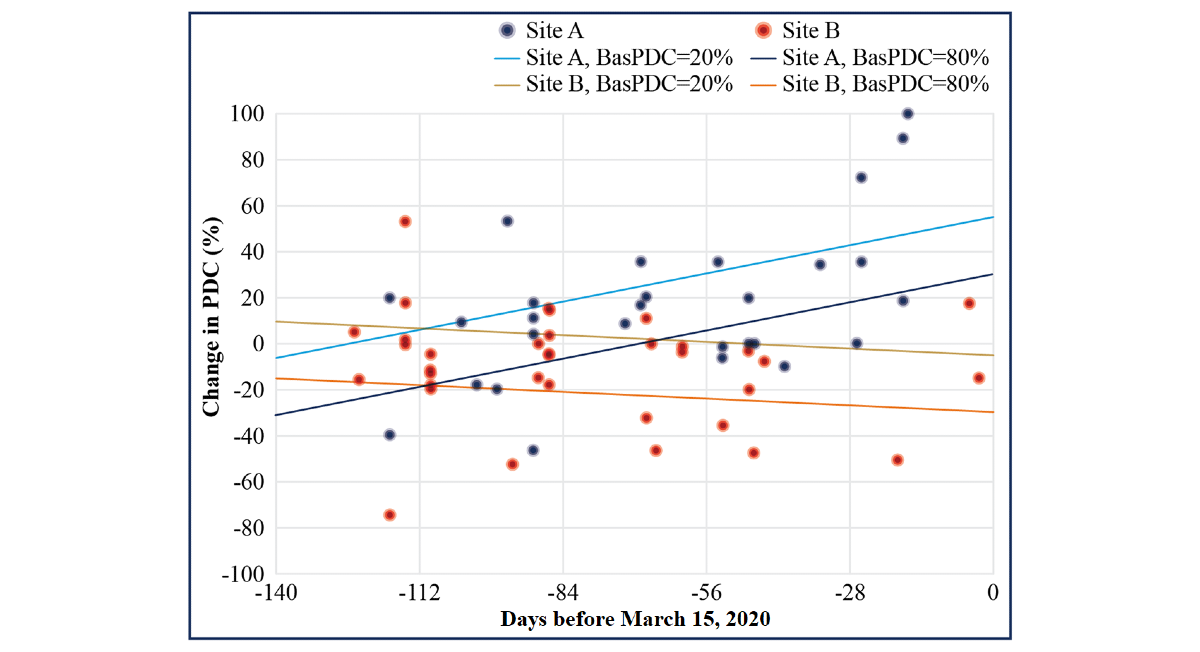
Proportion of days covered (PDC) change at 24 weeks of follow-up. PDC increases were observed at site A and PDC decreases were observed at site B, but a lower baseline PDC was associated with a higher PDC change at 24 weeks at both sites. The duration of time from March 15, 2020, to the end of each participant’s follow-up was associated with greater PDC increases at site A (where the lockdown duration after March 15, 2020, was shorter) and greater decreases at site B (where the lockdown duration after March 15, 2020, was longer). BasPDC: baseline proportion of days covered.

The mean increase in the PDC was 4.5% (*P*=.32) on comparing the first 12 weeks of follow-up to the baseline interval and was 1.5% (*P*=.70) on comparing 24 weeks of follow-up to the baseline interval. However, PDC changes differed between sites. Site A had a significant mean increase in the PDC (20.9% at 12 weeks; *P*=.003 and 16.7% at 24 weeks; *P*=.01). At site B, the mean PDC did not change significantly at 12 weeks (−8.2%; *P*=.14) but declined over 24 weeks (−10.3%; *P*=.02). Additionally, changes in the PDC were negatively correlated with baseline PDC (24 weeks: r=−0.55; *P*<.001; 12 weeks: r=−0.52; *P*<.001), reflecting mainly positive PDC changes at lower baseline PDC and mainly negative changes at the highest baseline PDC. Importantly, PDC change, adjusted for baseline PDC, varied with the proportion of the follow-up interval that occurred after March 15, 2020, but did so differently at the 2 sites. The PDC change from baseline through follow-up increased as the proportion of follow-up days after March 15, 2020, at site A increased, but decreased with an increasing proportion after March 15, 2020, at site B (Figure 3).

### Medication Adherence Relative to App Use

App use measured by the number of follow-up days on which the app was used at least once was not statistically significant (*P*=.46) when added to a model of PDC change that also included baseline PDC, time from March 15, 2020, to the end of each participant’s follow-up, site, and the interaction between site and time from March 15, 2020, to the end of follow-up. However, the indicator for increased app use after March 15, 2020, was statistically significant when it replaced app use over the follow-up interval in this model (Table 2). After adjusting for the other predictors, PDC change was 13.8% greater in those with increased app use after March 15, 2020. In other words, those with increased app use after March 15, 2020, showed either a smaller drop in the PDC or a greater gain in the PDC (depending on baseline PDC, site, and days after March 15, 2020), while those with decreased app use showed a greater reduction in the PDC where the PDC declined in both groups, or a smaller increase where it increased in both groups.

**Table 2:** Linear model of the change in the proportion of days covered from baseline to 24 weeks of follow-up.

Parameter^a^	Estimate	SE	*P* value
Intercept	4.4493	11.0926	.69
App use increased after March 15, 2020	13.7584	6.1096	.03
App use decreased after March 15, 2020	0	N/A^b^	N/A
Baseline PDC^c^	−0.3928	0.0862	<.001
Days from enrollment through March 15, 2020	−0.0116	0.1247	.93
Site A	53.3618	14.3925	<.001
Site B	0	N/A	N/A
Days from enrollment through March 15, 2020, at site A	−0.4695	0.1803	.01
Days from enrollment through March 15, 2020, at site B	0	N/A	N/A

^a^Model variables included baseline proportion of days covered, site, time from March 15, 2020, to the end of each participant’s follow-up, the interaction between site and time from March 15, 2020, to the end of follow-up, and an indicator for increased app use after March 15, 2020.

^b^N/A: not applicable.

^c^PDC: proportion of days covered.

### Qualitative Data

Eleven patients (mean age, 26.4 years; 64% [7/11] males; 100% [11/11] Black; 73% [8/11] HbSS; 45% [5/11] low app users) completed interviews across the 2 sites. Site B’s closure during COVID-19 had a greater impact on patients, who had difficulty obtaining hydroxyurea and reaching their providers and the clinic for nonurgent or emergent reasons. One low user from site B stated:

Before COVID-19, I could just call my clinic or doctor and ask if I could come in and it would be a ‘yes’, but now, its [COVID-19] cut down on the days the clinic is open and the time the clinic is open. It's harder to get in.

However, almost all reported that the *InCharge Health* app helped support more consistent daily medication use (Multimedia Appendix 2). One high user from site A stated:

I can appreciate it [the app]. It helped me. I think it’s a good thing. I think it makes me better with my hydroxyurea.

Consistent with patients, providers and administrators reported that clinic shutdowns during COVID-19 negatively impacted the ability to care for patients. For example, because fewer patients were coming to the clinic, there was a reduction in the need to use the *HU Toolbox* app as an aid for hydroxyurea prescribing (Multimedia Appendix 3). One provider from site B reported:

It [COVID-19] definitely impacted [app use]. As fellows, we were not coming to the clinic as often for at least two to three months. So, I didn't happen to think about the app or just didn't have an opportunity to use it.

Research staff at both sites also reported that reduced in-person clinic visits was a barrier for implementing the study in general. One staff member stated:

It has been quite difficult during the pandemic. It was easier for us when we were in person. We had that carved out time when [patients] weren't doing anything else, they were specifically focused on what we were doing.

## Discussion

Hydroxyurea is an evidence-based therapy in SCD, with proven clinical benefits, but its uptake is low. In a multicenter NHLBI-funded study, we tested the use of mHealth to improve hydroxyurea use among adolescents and adults with SCD. At the first 2 study sites, participants were exposed to the early phases of the COVID-19 pandemic, which included disruption in their regular SCD care. While the ubiquitous access to mobile technology among patients with SCD represents a unique opportunity to leverage mHealth interventions to support clinical care, the contextual changes, such as those during global emergencies, can affect its implementation. Our study is the first to assess, among individuals with SCD, the impact of the COVID-19 pandemic on the implementation of an mHealth behavioral intervention aimed at improving medication adherence. In the 2 clinical trial sites where study activities happened during the early phases of the pandemic, we found evidence of significant reductions in the implementation of the app relative to the duration of the clinic lockdown in response to the COVID-19 pandemic. While low baseline adherence levels predicted higher improvements in adherence, the pandemic disruptions also affected the adherence to hydroxyurea, which was proportionally reduced to the duration of the clinic lockdown. However, we also found evidence of the benefit of mHealth to improve adherence. Among patients whose mHealth use increased after the start of the lockdown, improvements in hydroxyurea adherence were also observed. Our findings highlight the influence of unplanned contextual changes on the implementation of mHealth behavioral interventions and the potential benefits of investing in strategies to sustain use. These data are key for the future implementation of mHealth behavioral interventions, for both patients and providers, in clinical settings during pandemics or other similar situations.

Earlier studies have demonstrated the potential efficacy of mHealth interventions for enhancing hydroxyurea adherence among patients with SCD [23,24]. It is worth noting that not receiving hydroxyurea, along with other factors, is predictive of mortality in SCD patients with COVID-19 infection [25], supporting the additional clinical benefits of hydroxyurea use, particularly during the pandemic. However, it is possible that the effect of mHealth may be mediated or moderated by ongoing contacts with health care providers, as demonstrated by the lower app use (and consequent lack of an effect for improving adherence) at site A, where disruptions in patient-provider contact were prolonged, and the higher app use at site B, where lockdown was shorter and telehealth was implemented quicker.

In our study, the usage of the *InCharge Health* app varied among patients. We also found different barriers to app implementation, especially those related to access to the health system, with fewer in-person clinic visits, and low contact between patients and providers, potentially reducing hydroxyurea adherence. Further, providers and administrators reported that the *HU Toolbox* app was not used often due to clinic lockdown, which may have contributed to reduced gains in the PDC. To conceptualize how the lockdown disruptions influenced mHealth use and consequently medication adherence, we described the influence of the lockdown on the different targets of the mHealth intervention (Figure 1B). This model is supported by our qualitative data that validate the disruptions in care leading to decreased patient-provider contact, decreased hydroxyurea prescribing, decreased patient and provider mHealth use, and decreased hydroxyurea adherence.

During the COVID-19 pandemic, reliance on telehealth exponentially increased, and for some chronic conditions, it not only facilitated care delivery but also improved health outcomes [26,27]. In our study, although mHealth may have supported adherence, this effect was moderated by the duration of the lockdown, which negatively impacted app use over time and consequently affected hydroxyurea adherence. The early use of telemedicine at site A might have helped support the use of the patient app, as it maintained patient-provider contact, and might have mitigated the clinical care disruptions. A full evaluation of the impact of telemedicine is planned when the final results of the trial are available.

The finding that reduced in-person visits was a barrier to study implementation was not surprising, as the impact of COVID-19 on clinical trials is well recognized [28]. Various strategies were suggested to mitigate some of these effects, such as (1) remote enrollment and follow-up and completion of study procedures when possible; (2) prioritization of primary outcomes; (3) alternative approaches for outcome assessment; (4) obtaining three or more phone numbers and email addresses for patients and relatives or friends; and (5) using different ways to contact patients and families, including text messaging, phone calls, emails, or social media [28]. Although these strategies have been reported, we were unable to document their impact on study implementation, as use of telehealth, for instance, was limited in the first year of the pandemic at one of the sites.

Our study has limitations. Data included in this analysis were from 2 study sites with relatively small sample sizes, which limits the generalizability of our findings. However, they do reflect the impact of the early institutional responses to the COVID-19 pandemic, which also occurred in other health institutions worldwide. Additionally, the results presented are not representative of the full study results, as this study is currently ongoing. Additional data regarding the effectiveness of mHealth for hydroxyurea adherence is, therefore, forthcoming. We also were not able to conduct interviews with all SCD participants to better understand specific barriers to hydroxyurea adherence during the pandemic, but our interview sample was purposefully selected based on the participants’ app use levels and achieved theme saturation. Because of the nature of the pandemic, we were not able to measure the mental health impact of the pandemic in the initial months and how it would have affected app use. Further, there are other possible variables beyond patient-level barriers or characteristics, such as system-level ones, that may have affected the implementation of our app during COVID, including clinics being shut down and limited use of telehealth at one site versus the other. Finally, although the PDC is an indirect measure of adherence, it is considered reliable and reflective of real-world settings (as opposed to adherence measured during clinical trials), and it has been used in many published research studies on SCD and other chronic medical conditions [29].

In conclusion, mHealth apps are promising tools for improving hydroxyurea adherence among adolescents and adults with SCD. In this preliminary analysis, we found significant impacts of the early clinic lockdowns, which reduced the implementation of the mHealth intervention for increasing hydroxyurea uptake. This disruption led to reduced patient adherence to hydroxyurea. However, disruptions to mHealth implementation were lower among participants who experienced shorter clinic lockdowns and among those who increased mHealth use during the pandemic, and evidence of the benefit was provided by higher hydroxyurea adherence. In qualitative analysis, we found concordance between low app use and perceived barriers to obtaining care early on during the pandemic. Triangulation of our findings suggests the benefit of mHealth for improving medication adherence and indicates that its use may be influenced by frequent contact with health care providers. Patients’ barriers to care access might have hindered app implementation, potentially reducing medication adherence. Investigation of added strategies to mitigate the effects of imposed care interruptions during major emergencies, particularly greater patient touchpoints (eg, patient coaching and health navigation), may “insulate” the implementation of interventions for increasing medication adherence. Future studies are essentially needed to better understand both patient- and system-level barriers in the context of pandemics or other similar situations. A focus on removing barriers to mHealth use during care disruptions will likely improve app implementation and medication adherence, ultimately reducing health inequities for vulnerable populations.

## Appendix

Multimedia Appendix 1 Interview questions to describe app effectiveness and implementation during COVID-19.

Multimedia Appendix 2 Thematic analysis of patient experiences with hydroxyurea adherence and health care access during COVID-19.

Multimedia Appendix 3 Thematic analysis of provider, administrator, and research staff experiences with app implementation during COVID-19.

Multimedia Appendix 4 Sickle Cell Disease Implementation Consortium Members.

## Abbreviations

mHealth: mobile health
NHLBI: National Health Lung and Blood Institute
PDC: proportion of days covered
RE-AIM: reach, effectiveness, adoption, implementation, and maintenance
SCD: sickle cell disease
SCDIC: Sickle Cell Disease Implementation Consortium
