# Supplementary material for: Impact of the COVID-19 Pandemic on the Implementation of Mobile Health to Improve the Uptake of Hydroxyurea in Patients With Sickle Cell Disease: Mixed Methods Study
Source: JMIR Form Res. 2022 Oct 14;6(10):e41415. doi: 10.2196/41415 (PMC9578525; doi:10.2196/41415)
Supplement: Multimedia Appendix 1 [file formative_v6i10e41415_app1.doc]

### Multimedia Appendix 1. Interview questions to describe app effectiveness and implementation during COVID-19.

| **RE-AIM Domain** | **Interview Question** | **Source** |
| --- | --- | --- |
|  |  |  |
| **Effectiveness** | How (if at all) has using the app changed the way you take hydroxyurea?  How (if at all) did COVID-19 change how you:  a. Got health care when you needed it (e.g., sheltering in place, worry about being infected, clinic changes due to COVID-19)?  b. Got your hydroxyurea (e.g., sheltering in place, transportation changes, clinic changes due to COVID-19)?  c. Took your hydroxyurea (e.g., feeling stressed, worry)? | Patients |
| **Implementation** | How (if at all) did COVID-19 impact your use of the app? | Providers |
|  | How (if at all) did COVID-19 impact implementation of the apps in your clinic? | Administrators |
|  | What were some of the specific challenges you encountered at your site during the trial related to COVID-19 (e.g., recruitment, implementation of the apps, level of interest in using the apps?)   1. What worked well? 2. What did not work well? 3. What else could have been done to support implementation of the apps at your site during the pandemic? | Research Staff |
