# Supplementary material for: Impact of the COVID-19 Pandemic on the Implementation of Mobile Health to Improve the Uptake of Hydroxyurea in Patients With Sickle Cell Disease: Mixed Methods Study
Source: JMIR Form Res. 2022 Oct 14;6(10):e41415. doi: 10.2196/41415 (PMC9578525; doi:10.2196/41415)
Supplement: Multimedia Appendix 2 [file formative_v6i10e41415_app2.docx]

### Multimedia Appendix 2. Thematic analysis of patient experiences with hydroxyurea adherence and health care access during COVID-19.

|  | Patient Representative Quotes | |
| --- | --- | --- |
| RE-AIM Domain | Site A | Site B |
|  |  |  |
| **Effectiveness** | I can appreciate it [the app], it helped me. I think it’s a good thing. I think it makes me better with my hydroxyurea. – High User  I had to plan my trips to the pharmacy for other times I had to leave the house so I’m not going out of the house so often. – Low User | Since I've started the app and I entered my medication on it, it's actually helped me to take my medication on time. – High User  Before COVID-19, every weekday, I could just call my clinic or doctor and ask if I could come in and it would be a “yes”, but now, it's cut down on the days that the clinic is open and the time the clinic is open. It's harder to get in. – Low User |
