# Supplementary material for: Impact of the COVID-19 Pandemic on the Implementation of Mobile Health to Improve the Uptake of Hydroxyurea in Patients With Sickle Cell Disease: Mixed Methods Study
Source: JMIR Form Res. 2022 Oct 14;6(10):e41415. doi: 10.2196/41415 (PMC9578525; doi:10.2196/41415)
Supplement: Multimedia Appendix 3 [file formative_v6i10e41415_app3.docx]

### Multimedia Appendix 3. Thematic analysis of provider, administrator, and research staff experiences with app implementation during COVID-19.

|  | Provider Representative Quotes | | | Administrator/Research Staff Representative Quotes | | |
| --- | --- | --- | --- | --- | --- | --- |
| RE-AIM Domain | Site A | Site B | Site A | | Site B |  |
|  |  |  |  | |  |  |
| **Implementation** | All our practices have been affected by COVID-19, not only hydroxyurea prescription or the app itself. Seeing patients has not been flowing very easily since COVID-19 and a lot of the telehealth visits would actually be more challenging in terms of keeping up with the patients’ care and their questions and their issues have kind of affected using the app obviously. –Low User | It definitely impacted [app use]. As fellows, we were not coming to the clinic as often for at least two to three months. So, I didn't happen to think about the app or just didn't have an opportunity to use it. – High User | It [COVID-19] really changed our priorities in terms of reaching out to our patients. We had fewer patients coming into the clinic. We made a concerted decision not to do significant dose escalation on hydroxyurea during that time period because we knew we would be limiting monitoring and so we were by far not as aggressive with our hydroxyurea management. Therefore, we [providers] weren’t utilizing the app. –Administrator | | It [implementation] has been quite difficult during the pandemic. It was easier for us when we were in person, which we had that carved out time that they [patients] weren't doing anything else, they were specifically focused on what we were doing. – Research Staff |  |
